# The genetic architecture of flowering time changes in pea from wild to crop

**DOI:** 10.1093/jxb/erac132

**Published:** 2022-04-06

**Authors:** Owen Williams, Jacqueline K Vander Schoor, Jakob B Butler, Stephen Ridge, Frances C Sussmilch, Valerie F G Hecht, James L Weller

**Affiliations:** School of Natural Sciences, University of Tasmania, Private Bag 55, Hobart, TAS 7001, Australia; School of Natural Sciences, University of Tasmania, Private Bag 55, Hobart, TAS 7001, Australia; School of Natural Sciences, University of Tasmania, Private Bag 55, Hobart, TAS 7001, Australia; School of Natural Sciences, University of Tasmania, Private Bag 55, Hobart, TAS 7001, Australia; School of Natural Sciences, University of Tasmania, Private Bag 55, Hobart, TAS 7001, Australia; School of Natural Sciences, University of Tasmania, Private Bag 55, Hobart, TAS 7001, Australia; School of Natural Sciences, University of Tasmania, Private Bag 55, Hobart, TAS 7001, Australia

**Keywords:** Adaptation, florigen, flowering time, *FT* genes, genetics, legume, pea, phenology, photoperiod, *Pisum*, QTL analysis

## Abstract

Change in phenology has been an important component in crop evolution, and selection for earlier flowering through a reduction in environmental sensitivity has helped broaden adaptation in many species. Natural variation for flowering in domesticated pea (*Pisum sativum* L.) has been noted and studied for decades, but there has been no clear account of change relative to its wild progenitor. Here we examined the genetic control of differences in flowering time between wild *P. sativum* ssp*. humile* and a typical late-flowering photoperiodic *P. s. sativum* accession in a recombinant inbred population under long and short photoperiods. Our results confirm the importance of the major photoperiod sensitivity locus *Hr/PsELF3a* and identify two other loci on chromosomes 1 (*DTF1*) and 3 (*DTF3*) that contribute to earlier flowering in the domesticated line under both photoperiods. The domesticated allele at a fourth locus on chromosome 6 (*DTF6*) delays flowering under long days only. Map positions, inheritance patterns, and expression analyses in near-isogenic comparisons imply that *DTF1*, *DTF3*, and *DTF6* represent gain-of-function alleles of the florigen/antiflorigen genes *FTa3*, *FTa1*, and *TFL1c/LF*, respectively. This echoes similar variation in chickpea and lentil, and suggests a conserved route to reduced photoperiod sensitivity and early phenology in temperate pulses.

## Teaser

Changes in flowering time following domestication of pea are attributable to four loci and include apparent gain-of-function variants of three florigen family genes.

## Introduction

Flowering time is an important adaptive trait which plays a crucial role in coordinating flowering to favourable seasonal conditions. In many cases it is strongly influenced by daylength and temperature, and this responsiveness is generated through complex regulatory pathways. For many species in the wild, strong requirements for specific environmental conditions impose strong constraints on phenology, and this has an adaptive benefit. However, from an agricultural perspective these requirements can often act as a physiological barrier to broader adaptation of crops outside their region of origin, and as a consequence have in many cases been relaxed through selection during domestication and subsequent diversification events (Gaudinier and Blackman, 2020). Well-documented examples are seen in the cereals, wheat and barley, where mutations in the *Ppd* and *Vrn* genes have adjusted photoperiod and vernalization sensitivity and have been linked to the expansion of these crops into northern Europe from south west Asia and Mediterranean regions (Cockram *et al.*, 2007). Similar adaptations are also seen during the evolution of legume crops, with a reduction in photoperiod sensitivity conferred by mutations in *PRR3*, *PHYA*, *E1*, and *GI* genes in soybean (*Glycine max*; Lin *et al.*, 2021), *PHYA3* and *COL2* in common bean (*Phaseolus vulgaris*; Weller *et al.*, 2019; Gonzalez *et al.*, 2021), and *ELF3* in pea (*Pisum sativum*), lentil (*Lens culinaris*), and chickpea (*Cicer arietinum*; Weller *et al.*, 2012; Ridge *et al.*, 2017), which have in each case contributed to expansion of their ecogeographical range.

The domesticated pea, *Pisum sativum*, is an important crop legume, with almost 36 Mt produced globally in 2019 (FAOSTAT, 2019), and was among the earliest plant species to be domesticated in the Neolithic period (Lev-Yadun *et al.*, 2000). Genetic and cytological analyses indicate that it most probably originated from the northern variety (var. *syriacum*) of the wild *P. sativum* ssp*. humile* (Ben-Ze’ev and Zohary, 1973; Jing *et al.*, 2010; Kreplak *et al.*, 2019), a quantitative long-day (LD) plant with a natural distribution ranging across Northeast Israel, Syria, South Turkey, and the Western side of the Zagros mountains in Iran (Zohary and Hopf, 1973). The earliest archaeological evidence for pea domestication is found in Çayönü in Turkey and Bouqras in Syria (Zohary and Hopf, 2000), and during its spread throughout Southern Eurasia it is inferred to have diverged into two distinct lineages (Jing *et al.*, 2010). An eastern expansion towards the Indian subcontinent and Himalayan region gave rise to the Afghanistan germplasm group, and the more prominent western expansion to Mediterranean Europe eventually gave rise to modern *P. s. sativum* cultivars. While these lateral expansions occurred relatively rapidly, expansion to higher latitudes in Eurasia appears to have been impeded by the maladaptive nature of the strong requirement for long photoperiods (Purugganan and Fuller, 2009; Weller *et al.*, 2012). The latitudinal expansion of *P. s. sativum* was presumably facilitated through selection for reduced photoperiod sensitivity, allowing a more reliable completion of the life cycle within the shorter summer growing season in cool-temperate regions, or under short photoperiods at lower latitudes. Early genetic studies employing the use of controlled short-day (SD) conditions to examine widely available natural variation for flowering time resulted in the discrimination of four loci: *EARLY* (*E*) on linkage group (LG) VI (Ps1), *STERILE NODES* (*SN*) on LGVII (Ps7), *LATE FLOWERING* (*LF*) on LGII (Ps6), and *HIGH RESPONSE* (*HR*) on LGIII (Ps5) (Murfet, 1971, 1973). The *HR* and *SN* loci have subsequently been identified as the circadian clock genes *ELF3a* and *LUX ARRHYTHMO* (*LUX*), respectively (Weller *et al.*, 2012; Liew *et al.*, 2014), and determine photoperiod sensitivity by delaying flowering under inhibitory (SD) photoperiods. The main functional variant at *HR* is widespread in the global pea germplasm and conditions the major difference between winter and spring growth habit (Weller *et al.*, 2012). In contrast, *sn* mutations eliminate photoperiod sensitivity completely, occur at much lower frequency, and most probably arose much more recently (Liew *et al.*, 2014). *LF* is a co-orthologue of Arabidopsis *TERMINAL FLOWER 1* that suppresses flowering under both SDs and LDs in proportion to its expression level, and has numerous naturally occurring alleles with variable dominance and ability to delay flowering (Murfet, 1975; Foucher *et al.*, 2003). The *E* locus has not been characterized at the molecular level but has been shown to promote flowering without altering photoperiod sensitivity more generally (Murfet, 1985). Although these four loci are well established, their relative importance in determining the differences in flowering time and photoperiod sensitivity between wild and domesticated material has not been examined. It is also not clear whether additional loci might also contribute to these differences.

In a previous study, we investigated the difference in photoperiod sensitivity between a wild line and a standard late-flowering domesticated accession by genetic analysis of flowering time in non-inductive SD conditions (Weller *et al.*, 2012). This study found two major (i.e. >15% variation explained) quantitative trait loci (QTLs) in positions consistent with identities as *HR* and *E*, but substantial residual variation in flowering time observed in the population also indicated the presence of additional undetected minor loci. Here we have built on this work to conduct a more thorough genetic analysis of flowering time variation between wild and domesticated *P. s. sativum* using an F_8_+ recombinant inbred population and a high-density linkage map. Our results clarify the importance of *HR* and *LF* loci, provide new understanding of *DTF1/E*, and identify a new locus, *DTF3*, influencing flowering time under both LDs and SDs. Candidate analysis indicates that the *DTF1/E* and *DTF3* loci probably represent gain of function associated with *FT* genes.

## Materials and methods

### Plant material, growing conditions, and phenotypic evaluation

An F_8_+ recombinant inbred line (RIL) population of 138 lines derived from the F_2_ interspecific cross between the wild *P. s. humile* ‘type’ line (JI1794) and a domesticated *P. s. sativum* cultivar (NGB5839) was developed using single seed descent under LD glasshouse conditions as previously described in Weller *et al.* (2012). The phenotyped populations were grown in a glasshouse under controlled LD or SD photoperiod conditions (LD=16 h light and 8 h dark, SD=8 h light and 16 h dark) with four replicate plants for each individual RIL. These were sown in 14 cm pots prepared with a 1:1 gravel:vermiculite mixture, and covered with a 3 cm layer of sterilized potting mix which included controlled-release fertilizer. All plant material was supplied with sufficient water and nutrients. Plants were grown two per pot to maximize use of the space available, a standard practice previously shown not to cause significant detriment to plant growth or alteration to phenology.

Flowering traits assessed for each plant were (i) node of flowering initiation (NFI) as the number of nodes on the main stem to the first flower, (ii) days to flower (DTF) as the number of days between seedling emergence and the day of opening of the first flower; and (iii) reproductive nodes (RNs) as the total number of floral nodes of the main stem. Significant variation in each trait was determined using Tukey’s HSD pairwise analysis (*P*<0.05).

### DNA extraction and genotyping

Genomic DNA was extracted from young leaflets using the cetyltrimethylammonium bromide (CTAB) extraction protocol (Doyle and Doyle, 1990). The RIL population and parental lines were genotyped by Diversity Array Technology Pty. Ltd (Canberra, Australia) using DArTseq markers (Kilian *et al.*, 2012) which were supplemented with 24 gene-based anchor markers previously generated for chromosomes 1, 3, 5, 6, and 7 (Supplementary Table S1). Markers were assigned into four quality classes (or excluded from analysis) based on (i) call rate, (ii) reproducibility, (iii) segregation distortion, and (iv) proportion of heterozygotes, to assist in map curation.

### Linkage map construction and synteny assessment

To assist with map construction, a marker binning process was employed using SimpleMAP (Jighly *et al.*, 2015), with markers grouped into bins based on a recombination threshold of four (to span <3 cM). A representative marker from each bin was then selected and used to create a skeleton linkage map using JoinMap v4.0 (Van Ooijen, 2006). Markers were assigned into seven LGs using JoinMAP at a logarithm of odds (LOD) threshold of five. Markers were ordered within LGs using the Kosambi mapping function (Kosambi, 1943) and the maximum likelihood algorithm, and contrasted to orders generated using the regression algorithm. Regions with conflicting marker ordering between algorithms or high segregation distortion were resolved by stringent post-mapping marker exclusion based on marker quality. Markers placed into bins earlier were then integrated into the skeleton map around their respective representative marker and ordered based on recombinations. LGs were ordered and numbered, initially according to their classical designation, and subsequently according to their corresponding chromosomes (Kreplak *et al.*, 2019). Maps were visualized using MapChart (Voorrips, 2002), and mapping quality was assessed by plotting pairwise recombination fraction and LOD values as a heat map using Rqtl (Broman *et al.*, 2003).

The linkage map was assessed for synteny against the *P. sativum* reference genome (v1a, Kreplak *et al.* 2019) and the related reference genomes *Medicago truncatula* (v4.0, Tang *et al.*, 2014), *Cicer arietinum* (v2.0, Parween *et al.*, 2015), *Lens culinaris* (v1.0, Bett *et al.*, 2016), and *Trifolium pratense* (v2.0, Ištvánek *et al.*, 2014), using Geneious V.9.1.2 (http://www.geneious.com). Potential orthologous positions were determined by BLAST searches of marker sequences with the following parameters: Map multiple best matches: none; Trim paired reads; Minimum support for structural variant discovery: 2 reads; Allow gaps set to a maximum of 5% per read and a maximum gap size of 3; Word length: 6; Index word length 6; Maximum mismatch per read: 35%; and Maximum ambiguity: 5. Fine tuning: none. Chromosome/LG lengths were standardized before visualizing synteny via Marey plots.

### QTL analysis

QTL analysis for flowering time was performed using MapQTL v6 (Van Ooijen, 2009) on the linkage map that had been thinned of every third marker to increase computational efficiency. In brief, QTLs were defined by a >3 LOD score and identified using the interval mapping (IM) function, followed by Automatic Cofactor Selection (ACS). Iterative searches for additional QTLs were performed using the Multiple QTL Model (MQM) function, which increases the power of QTL analysis by reducing residual variances attributed to previously identified QTLs (cofactors). The amount of variation explained by each QTL was estimated using the coefficient of determination (*R*^2^) which is represented as the phenotypic variance explained (PVE). Those QTLs with a PVE score >15% were considered as major and those with <15% as minor.

The genomic location of QTLs and other markers in this study will be referred to by the *P. sativum* LG positions reported in this study to allow comparisons with both historic *P. sativum* linkage maps and the recently released reference genome (Kreplak *et al.*, 2019).

### Advanced-generation segregating populations

To verify the effect and refine the placement of these QTL regions, F_3_–F_6_ populations were developed from segregating individuals from the original F_2_ population, and selected by genotyping for markers at or around the QTL peaks (Supplementary Table S2). After verifying that the QTL effect was segregating, putative candidate genes for flowering time were identified within the refined region.

### Gene expression

In experiments examining whether QTL effects were associated with altered regulation of underlying candidate genes, leaflets from the uppermost fully expanded leaf and apical buds were harvested and frozen in liquid nitrogen. Total RNA was extracted using the Promega SV Total RNA Isolation System (Promega, Madison, WI, USA) and RNA concentrations were quantified using a NanoDrop™ 8000 spectrophotometer. Reverse transcription was conducted using Tetro Reverse Transcriptase (Bioline, Meridian Bioscience) in a final volume of 20 μl with 1 μg of total RNA according to the manufacturer’s instructions. A negative control without reverse transcriptase was routinely included to monitor genomic DNA contamination. First-strand cDNA was diluted five times and 2 μl was used in each real-time PCR. Quantitative reverse transcription PCRs (qRT–PCRs) were performed in a Rotor-Gene Q thermocycler with Rotor-Gene 6 Version 6.1 (Qiagen) using the SensiFAST™ SYBR kit (Bioline, Meridian Bioscience). Each biological replicate (*n*=3–4) consisted of pooled material from two plants, and was represented in qPCR analysis by two technical replicates, which were averaged to provide a single value for the biological replicate/sample. Relative transcript levels were evaluated against the *ACTIN* reference gene, previously shown by Hecht *et al.* (2011) to be stably expressed at a uniform level in comparable tissue harvests taken from different flowering genotypes. Significant variation in expression levels of candidate genes was determined using a Tukey’s HSD pairwise analysis (*P*<0.05). Primer sequences are given in Supplementary Table S3.

## Results

### Linkage map construction and synteny

Genotyping of the JI1794×NGB5839 RIL population (*n*=138) identified 6000 DArTseq markers, of which 1214 were selected for mapping after filtering and binning, and were supplemented with 24 gene-based PCR markers (Supplementary Table S1) to assist with map orientation and gene targeting. These were employed to create a skeleton map of 905 markers after post-mapping marker exclusion. After reintegrating the non-mapped bin markers, a finalized linkage map consisting of 4599 markers (4575 DArT and 24 anchor markers) spanning a total length of 1617 cM was achieved (Supplementary Tables S4, S5). The mean marker distance was 0.35 cM, with the largest gap of 5.8 cM on LGVII.

A comparison between this high-density linkage map and the *P. sativum* genome assembly (Kreplak *et al.*, 2019) revealed a high level of inferred synteny (93.1%) and collinearity across the seven LGs, with 94.0% of markers mapping to the genome (Supplementary Fig. S1). Minor mapping variations were observed at the start of LGIV (Ps4) and the midsection of LGVII (Ps7), but may reflect limitations of the scaffolding approach used in genome assembly (Kreplak *et al.*, 2019). Areas of reduced mapping resolution were observed in LGV (Ps3), LGI (Ps2), and LGVI (Ps1), probably indicative of regions with either suppressed recombination or low gene density (as DArTseq marker have a gene bias).

We also compared orthologous positions of all markers against the smaller and more complete genomes from *Medicago truncatula* (Supplementary Fig. S2) and *Cicer arietinum* (Supplementary Fig. S3) where our results strongly corresponded to previous detailed syntenic comparisons (Duarte *et al.*, 2014; Tayeh *et al.*, 2015; Ma *et al.*, 2017), with LGII (Ps6) exhibiting numerous inversions and rearrangements.

We also considered this a good opportunity to compare the syntenic relationship of the closely related lentil (*Lens culinaris*; Supplementary Fig. S4) and red clover (*Trifolium pratense*; Supplementary Fig. S5). While a lower percentage of our markers could be mapped in lentil (59.6%), particularly at the centre of LGIV (Ps4)/Lc7 and LGVI(Ps1)/Lc2, overall a high level of inferred synteny (72%) was found. Furthermore, a high level of collinearity was found across all chromosomes apart from on LGII (Ps6) where, as for the *M. truncatula* comparison, numerous inversions and rearrangements were seen. Translocation events were found on LGI (Ps2), LGII (Ps6), and LGIII (Ps5), with the translocation on Ps5 also similar to that in *M. truncatula*. There is another known complex translocation event on LGVI (Ps1) in other legume species (Kaló *et al.*, 2004; Tayeh *et al.*, 2015), but this was not observed here. For red clover, despite a high proportion of markers which could be mapped (82.9%), the comparative analysis showed a lower degree of synteny than expected (30.1%), possibly due to this genome assembly being less complete.

### QTL analysis for flowering time traits

**Fig. 1.**
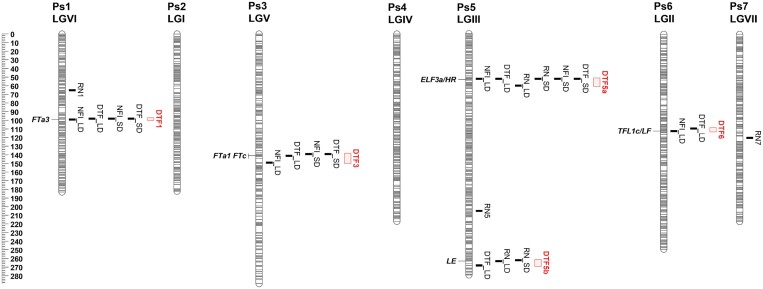
Linkage map showing QTLs detected in the JI1794×NGB5839 RIL population. QTL nomenclature follows Table 1. Scale is cM.

Several QTLs were identified in this RIL population for each of the flowering-related traits assessed, and in many cases QTLs for different traits were found to co-locate. A total of five QTLs for DTF were identified across both conditions (Fig. 1; Table 1). Three of these, on chromosomes 1, 3, and 5, were detected in both LDs and SDs, and are hereafter referred to as *DTF1*, *DTF3*, and *DTF5a*. All three co-located with QTLs for NFI, and *DTF5a* also co-located with a QTL for RN. Another co-locating QTL for DTF and NFI (*DTF6*) was identified on Ps6, but only in LD conditions. A final co-locating QTL for DTF in LDs and RN in both growing conditions was found in a location on Ps5 distant from *DTF5a*, and hereafter is referred to as *DTF5b*. Several minor QTLs for RN were also detected in other regions (Fig. 1; Table 1). In terms of flowering time, domesticated alleles at *DTF1*, *DTF3*, and *DTF5a* all contributed to early flowering, whereas the domesticated alleles at *DTF5b* and *DTF6* were associated with later flowering.

**Table 1.** Details of QTLs for flowering time

QTL	Chr/LG	Trait	Photoperiod	Map Position (cM)	Genome position (bp)	PVE (%)	LOD	Peak marker	Early flowering genotype
*RN1*	Ps1/VI	RN	SD	64.621	109270523–109270581	7.5	5.03	3554423_3	
*DTF1*	Ps1/VI	DTF	LD	85.955	169181425–169181467	19.1	9.98	3556333_2	Domesticated
		DTF	SD	84.678	167809482–167809413	18.3	17.31	3565843_3^*a*^	
		NFI	LD	84.678	167809482–167809413	31.0	15.34	3565843_3^*a*^	
		NFI	SD	84.678	167809482–167809413	30.5	20.24	3565843_3^*a*^	
*DTF3*	Ps3/V	DTF	LD	138.464	190458976–190459045	5.5	3.99	3545005_1	Domesticated
		DTF	SD	149.281	295854961–295855030	3.8	4.43	3548055_1^*a*^	
		NFI	LD	135.48	199418986–199419055	6.3	4.33	3642241_3	
		NFI	SD	149.281	295854961–295855030	3.5	3.19	3548055_1^*a*^	
*DTF5a*	Ps5/III	DTF	LD	51.618	66636624–66636693	12.6	6.47	3564019_4	Domesticated
		DTF	SD	51.618	66636624–66636693	59.5	37.27	3564019_4	
		NFI	LD	51.618	66636624–66636693	9.3	5.38	3564019_4	
		NFI	SD	51.618	66636624–66636693	39.0	24.04	3564019_4	
		RN	LD	60.309	138847091–138847151	15.5	7.47	3548887_2	
		RN	SD	51.659	68261427–68261482	36.9	18.39	4663518_2	
*RN5*	Ps5/III	RN	SD	205.351	468488251–468488318	4.4	3.00	5251991_3	
*DTF5b*	Ps5/III	DTF	LD	267.940	566189364–566189433	6.8	3.54	4661775_2	Wild
		RN	LD	262.876	566189364–566189433	18.0	8.54	4661529_2	
		RN	SD	261.535	564000472–564000403	7.8	5.08	3569442_1	
*DTF6*	Ps6/II	DTF	LD	112.305	113510652–113510585	6.2	4.4	4657639_1	Wild
		NFI	LD	112.305	113510652–113510585	9.4	5.42	4657639_1	
*RN7*	Ps7/VII	RN	LD	120.387	199885262–199885221	11.8	5.84	3544432_3	

RN, number of reproductive nodes at maturity; LG, linkage group; DTF, days to first open flower; NFI, node of flower initiation; SD, short days; LD, long days; PVE, proportion of variance explained; LOD, logarithm of odds.

^*a*^ The BLAST location of the original peak marker sequence was to a non-equivalent chromosome in the *Pisum sativum* genome assembly, so the next closest marker with correct positioning is reported.

**Fig. 2.**
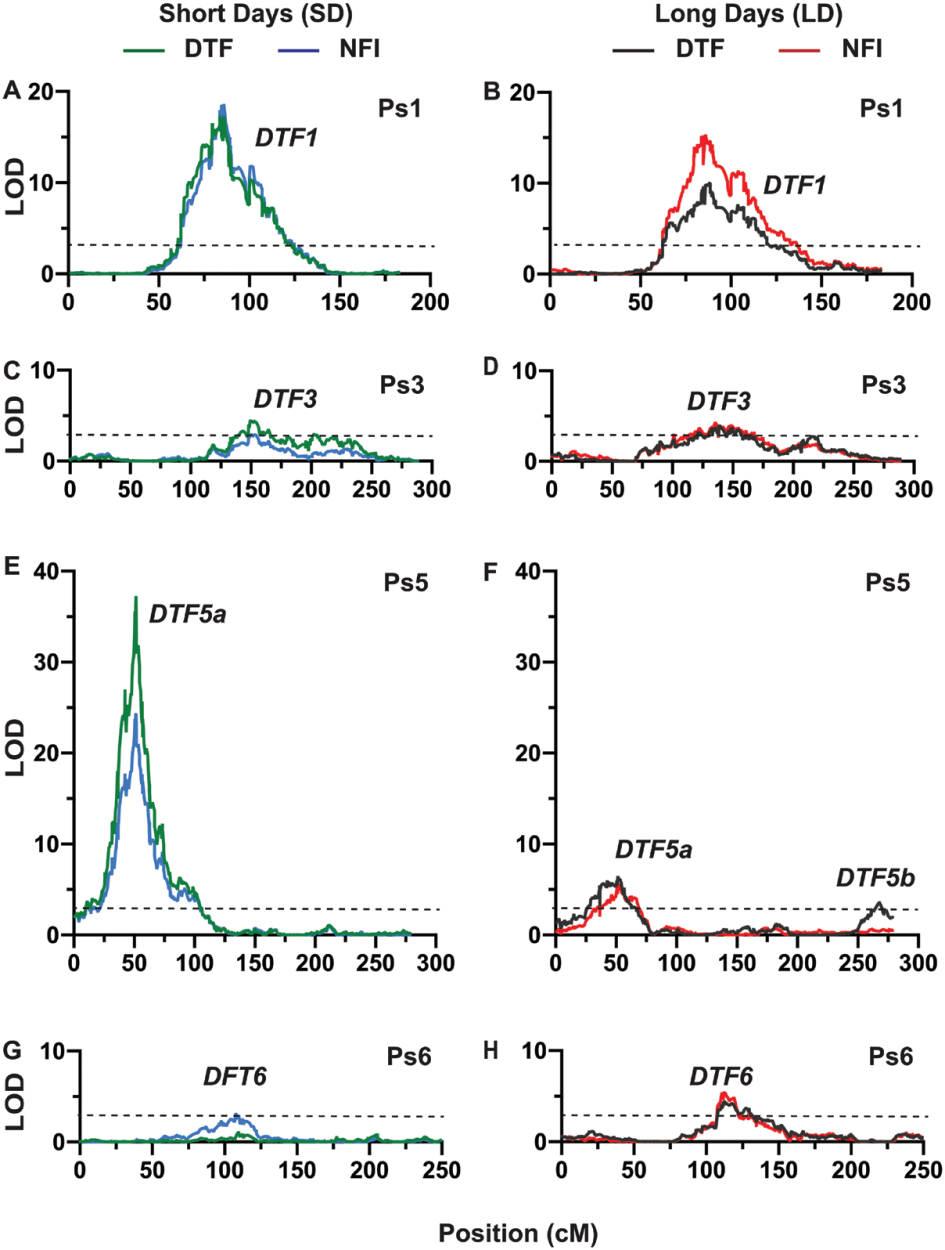
QTLs for flowering time detected under short or long days. Each plot shows LOD score against linkage group location for flowering time (days to flower, DTF) or the node of flower initiation (NFI) under short- (A, C, E, G) or long- (B, D, F, H) day conditions. Dashed horizontal lines represent a significance threshold of LOD score of 3.

The equivalence of *DTF5a* to the *HR* locus, its identity as *PsELF3a*, and its effects on both flowering and maturity traits have been previously established (Weller *et al.*, 2012). Consistent with this and other previous reports (Murfet, 1973), its effects were stronger under SDs where it explained 60, 39, and 37% of the phenotypic variation for DTF, NFI, and RN, respectively (Table 1; Fig. 2E; Supplementary Fig. S6). However, it also made a smaller but nevertheless significant contribution to variation in LDs, controlling 13, 9, and 16% of the variation in LDs for these same three traits (Table 1; Fig. 2F; Supplementary Fig. S6).

*DTF5b* was detected in LD conditions only and explained 7% of the variation in flowering time, but was found for RN in both day lengths, explaining 8% and 18% of the phenotypic variation in SDs and LDs, respectively (Table 1; Fig. 2F; Supplementary Fig. 6A). It was located close to Mendel’s *LE* locus, a well-known major regulator of gibberellin biosynthesis and modulator of plant growth and development (Lester *et al.*, 1997), in both the linkage map (3.3 cM) and the genome (~1 Mb). Given that the domesticated parent NGB5839 carries an induced mutation at Mendel’s *LE* locus (Lester *et al.*, 1999), segregation was expected in this population and was clearly evident in variation for plant height, and it seems reasonable to conclude that *DTF5b* is equivalent to *LE*.

*DTF1* has also been identified previously as a major flowering QTL in SD conditions (QTL6; Weller *et al.*, 2012) and was considered as likely to be equivalent to the *E* locus (Murfet, 1971). In the present study, *DTF1* explains 18% and 31% of the variation for DTF and NFI, respectively, under SDs (Table 1; Fig. 2A). It also explains a similar proportion of variation under LDs, 19% for DTF and 31% for NFI (Table 1; Fig. 2B), suggesting that its effects are not closely related to photoperiod sensitivity. Of the two other QTLs for flowering time identified in the RIL population, *DTF6* explains 6% and 9% of the variation for DTF and NFI, respectively, in LDs, but was below threshold in SDs (Table 1; Fig. 2G, H). *DTF3* explains 6% of the flowering time variation for both traits in LDs, and 4% for both traits in SDs (Table 1; Fig. 2C, D).

### Individual effects and interaction of flowering time QTLs in the RIL population

**Fig. 3.**
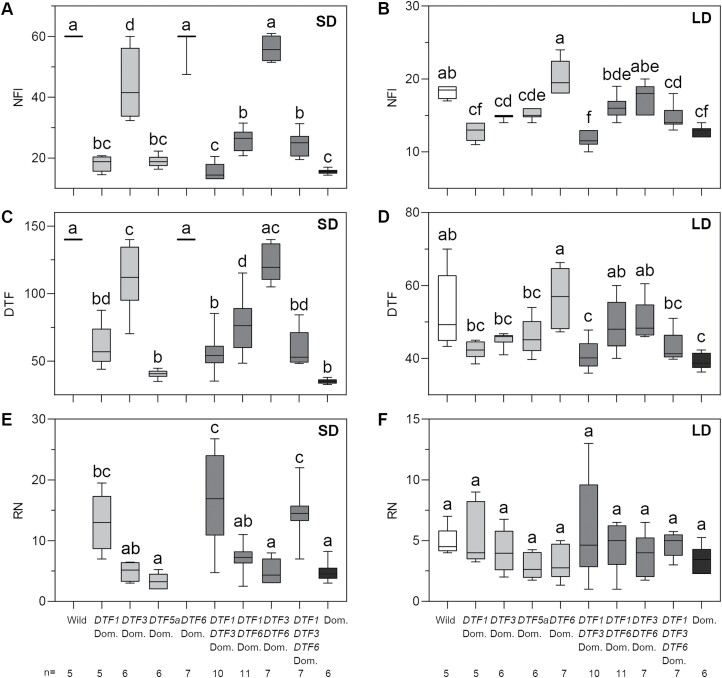
Effects and selected interactions of flowering time QTLs in the RIL population. The RIL population was classified according to genotypes at the four flowering time QTLs detected. The data shown represent classes homozygous for wild or domesticated (Dom) alleles at all four loci together with selected classes comprised of homozygous for domesticated alleles at one or more loci as indicated. (A, B) Node of flower initiation (NFI); (C, D) days to flower (DTF); (E, F) number of reproductive nodes at apical arrest (RN) shown for plants in long-day (LD) or short-day (SD) conditions. The number of lines in each class (*n*) is indicated below (E) and (F). The experiment was terminated at 140 d after sowing, at which time plants in the wild and *DTF6* Dom classes had not developed open flowers under SD conditions, and nominal minimum values of 140 d/60 nodes are shown in (A) and (C).

We next used the genotype of the peak marker for each QTL to categorize genotypic classes within the RIL population, and examine the effects of each QTL individually under the two different photoperiod conditions. The results confirm the importance of *DTF1* and *DTF5a* for the control of NFI and DTF under SDs, as the presence of either domesticated allele was associated with early flowering that did not differ in flowering node or time from the domesticated line NGB5839 (Fig. 3A, C; *P*>0.05). Similarly, in a domesticated background with respect to other flowering time QTLs, the presence of the wild allele at both loci resulted in a plant that flowered similarly to the wild parent (Fig. 3A, C; *P*>0.05). In LD conditions, substitution of the domesticated *DTF1* allele had the strongest individual effect, with no significant difference in NFI or DTF from the fully domesticated genotype (Fig. 3B, D). Plants carrying only the *DTF3* domesticated allele flowered at an intermediate time significantly different from wild and domesticated parental genotypes, in both SDs and LDs (Fig. 3A, D). The influence of the domesticated allele of *DTF6* alone in an otherwise wild genetic background was not detectable under SDs, which was as expected given that the wild genotype did not flower under these conditions. However, its effect was clear in the presence of domesticated alleles at one or more of the other loci (Fig. 3A, B).

### Individual QTL effects are confirmed in near-isogenic material

**Fig. 4.**
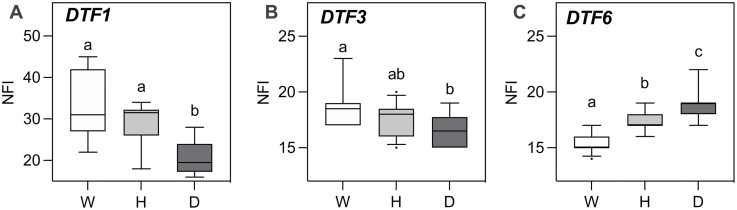
Validation of flowering time QTLs in advanced-generation segregating populations. Data represent the node of flower initiation (NFI) under long-day conditions in F_4_/F_5_ progenies of the JI1794×NGB5839 cross selected to segregate for the target loci (A) *DTF1*, (B) *DTF3*, and (C) *DTF6*. The three genotypic classes are indicated; homozygous wild (W), heterozygous (H), homozygous domesticated (D). In each panel, values not significantly different in a one-way ANOVA with Tukey’s multiple comparison test are indicated by the same letters.

Of the five loci identified, we focused on the three that were less well understood (*DTF1/E*, *DTF3*, and *DTF6*) for further investigation. In order to validate their effects and refine their map positions as a basis for identification of candidate genes, we developed advanced-generation segregating populations from the original F_2_, which was genotyped previously (Weller *et al.*, 2012). We fixed the majority of QTLs as the wild allele where possible and selected heterozygosity for the target QTL. Supplementary Table S2A provides details of these populations, which were grown in LD conditions, genotyped for relevant peak markers, and scored for flowering time (Fig. 4).

An advanced F_5_ segregating population of 32 individuals was developed for *DTF1*, fixing the other flowering QTLs for the late allele (domesticated for *DTF6* and wild for *DTF5a* and *DTF3*; Supplementary Table S2A). On this genetic background, plants carrying the domesticated allele of *DTF1* flowered earlier than those with the wild allele by ~10 nodes (Fig. 4A). Interestingly, although a co-dominant effect of *DTF1* was reported in the analysis of the original F_2_ under SDs (Weller *et al.*, 2012), under LDs the *DTF1* heterozygotes did not differ significantly from the wild homozygotes.

For *DTF3*, 45 plants of an F_4_ advanced segregating population was also developed (Supplementary Table S2A) and genotyped for the *DTF3* peak marker. This population contained all the other QTLs fixed as wild alleles to remove their influence on the phenotype. Consistent with observations in the RIL population, plants carrying the domesticated allele of *DTF3* flowered significantly earlier (*P*<0.015) than those carrying the wild allele (Fig. 4B). In the case of the *DTF6* population (*n*=85; Supplementary Table S2A), plants carrying the domesticated allele flowered significantly later than those with the wild allele (*P*<0.0001) (Fig. 4C), again consistent with the result from the QTL analysis. Interestingly, plants heterozygous for the domesticated allele also flowered significantly later than those with the wild allele, but earlier than those with the domesticated allele (*P*<0.0001 in both cases).

### Identifying and evaluating candidate genes for flowering QTLs

**Fig. 5.**
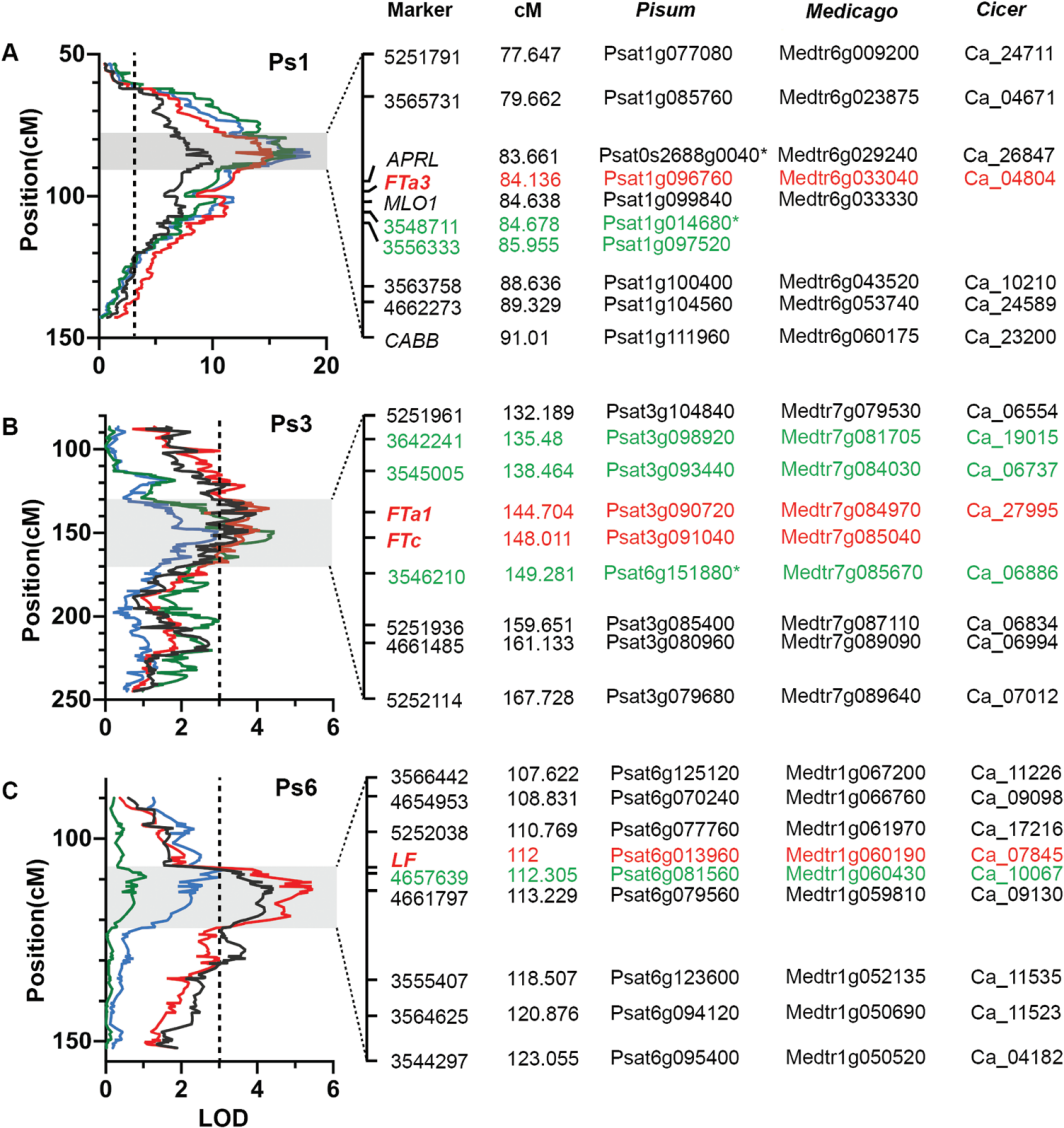
Identification of candidate genes under flowering time QTLs. Markers spanning the QTL peaks for *DTF1* (A), *DTF3* (B), and *DTF6* (C) were located in the pea genome and candidate flowering time genes in the intervals were identified. The presence of orthologous genes in the syntenic regions of Medicago and chickpea were also confirmed. In all three panels, LOD scores for DTF (SD), NFI (SD), DTF (LD), and NFI (LD) are represented by green, blue, black, and red lines, respectively. QTL peak markers are shown in green text, and candidate genes in red.

**Fig. 6.**
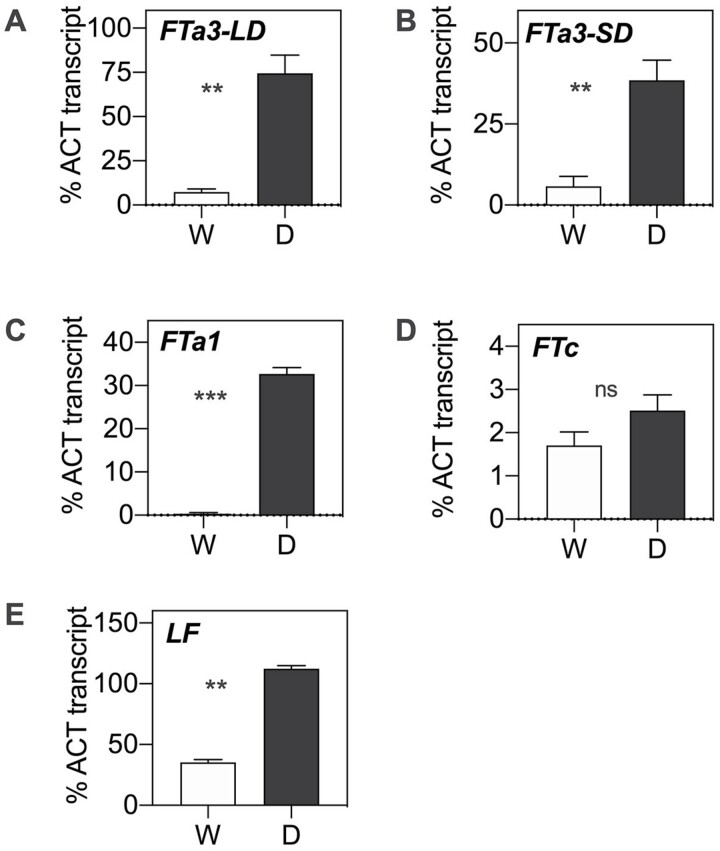
Effect of individual QTLs on expression of the corresponding candidate genes. (A, B) *FTa3* expression in leaves of 4-week-old plants of F_6_ near-isogenic lines (NILs) for *DTF1* grown under LDs (A) and 10-week-old plants grown in SDs (B). (C, D) Expression of *FTa1* in leaves of 3-week-old plants of F_5_ NILs for *DTF3* grown under LDs (C) and *FTc* in shoot apices of 4-week-old plants (D). (E) *LF* (*TFL1c*) expression in shoot apices of 4-week-old plants of F_5_ NILs for *DTF6* grown under LDs. W, wild allele; D, domesticated allele. In all panels, *n*=3–4 and *t*-tests indicated genotype values significantly different (*P*<0.01) except for (D) (*P*>0.1).

Two peak markers were identified for *DTF1*, one for DTF in LDs and another for the other flowering traits, both of which mapped to the middle of Ps1/LGVI between 84.6 cM and 86.0 cM (Table 1). However, their respective position on the genome spanned >2 Mb, so in order to narrow the candidate region a large F_4_ population of 396 individuals was grown and new markers (Supplementary Table S1) in the region were specifically designed for fine mapping. After progeny-testing several individuals in the F_5_ to confirm their genotype at *DTF1*, segregation analysis with these markers located *DTF1* close to the *MLO* marker and co-segregating with another florigen gene, *FTa3* (Fig. 5A; Supplementary Fig. S7, Supplementary Table S6). This gene is previously undescribed in pea but its presence in other temperate legumes has been noted (Ortega *et al.*, 2019). Sequencing of the *FTa3* coding sequence did not reveal any potentially causal mutation within the coding sequence, and *FTa3* expression was analysed in advanced-generation material near isogenic for the *DTF1* allelic difference (Supplementary Table S2B). In both LD and SD conditions, plants carrying the domesticated allele of *DTF1* were found to have a significantly higher expression level of *FTa3* in comparison with plants carrying the wild allele (*P*<0.005; Fig. 6A, B), consistent with *FTa3* as a potential causal gene underlying *DTF1*.

*DTF3* is less well defined positionally than the other flowering QTLs identified in this study, with each of the three flowering traits mapped in this region showing a different peak marker (Table 1). These markers all map on Ps3/LGV between positions 135 cM and 150 cM (Fig. 5B), and BLAST to positions near a cluster of florigen (*FT*) genes that is conserved in several different temperate legume species including *M. truncatula* (Laurie *et al.*, 2011) and chickpea (Ortega *et al.*, 2019). In pea, two genes map to the cluster and have been defined in the current genome version; *FTa1* (Psat3g090720) and *FTc* (Psat3g091040). *FT* genes play an important role as positive flowering regulators, with all three recognized subclades (*FTa*, *FTb*, and *FTc*) shown to promote flowering in transgenic Arabidopsis (Hecht *et al.*, 2011). As both *FTa1* and *FTc* have the potential to promote flowering, we compared their expression in near-isogenic lines for *DTF3* (Supplementary Table S2B) in leaf and shoot apex tissue, respectively, according to their previously described expression patterns (Hecht *et al.*, 2011; Laurie *et al.*, 2011). Consistent with their earlier flowering behaviour, plants carrying the domesticated allele showed statistically significant elevated expression of *FTa1* in leaf tissue (*P*<0.0001) (Fig. 6C) while the expression of *FTc* was not significantly different (*P*>0.1) between plants carrying domesticated or wild alleles of *DTF3* (Fig. 6D).

*DTF6* maps in the middle of Ps6/LGII around 112 cM (Table 1; Fig. 1), and details of the markers in the region are presented in Fig. 5C. BLAST searches with marker sequences specified a genomic location near the *LF/TFL1c* gene, well known for its role in inhibition of flowering (Murfet, 1975; Foucher *et al.*, 2003). Expression analysis of this gene showed that plants carrying the domesticated allele at *DTF6* had a significantly higher expression level compared with plants carrying the wild allele (*P*<0.002) (Fig. 6A), consistent with their later flowering.

## Discussion

Wild *P. sativum* subspecies *elatius* and *humile* do not flower under SD conditions <12 h, and this obligate LD requirement has been carried through into a subset of the domesticated germplasm (Weller *et al.*, 2012). From this extreme, variation within the domesticated germplasm extends to complete day neutrality, with accessions that flower as early in SDs as in LDs (Murfet, 1985). Detailed genetic analyses of different phenological classes among domesticated pea germplasm distinguished four loci contributing to this variation (Murfet, 1971, 1973), of which three have subsequently been characterized at the molecular level; *LF* (Foucher *et al.*, 2003), *HR* (Weller *et al.*, 2012), and *SN* (Liew *et al.*, 2014). In this study we revisited this question by considering differences between *P. s. humile*, the presumed wild ancestor of domesticated var. *sativum*, and a cultivar with an intermediate, quantitative photoperiod response commonly used as a reference line in genetic studies. The goal was to clarify the genetic architecture of flowering time differences captured in this evolutionary snapshot and to explore in more detail the nature and genomic position of component loci in relation to those identified previously in studies within the domesticated genepool.

Among the five QTLs identified, three almost certainly correspond to previously described flowering time loci. The *DTF5a* locus was the only one to show a strong photoperiod specificity, with a much stronger contribution under SDs than LDs (Table 1), and represents the previously characterized *HR/ELF3a* gene (Fig. 1) (Weller *et al.*, 2012). Map positions of two other QTLs, *DTF1* and *DTF6*, suggest that they may correspond, respectively, to the previously described *E* and *LF* loci (Murfet, 1971, 1975). *LF* has been identified as *TFL1c*, a subfunctionalized co-orthologue of Arabidopsis *TFL1* that has retained effects on flowering time but not on inflorescence determinacy, and several deletion and substitution mutants indicate its clear role in the inhibition of flowering (Foucher *et al.*, 2003). However, an extensive allelic series at *LF* has been reported, including putative gain- and loss-of-function alleles, and a survey of sequence diversity across various *LF* allelic variants could not locate changes within the coding region for some alleles. This suggests that the locus might be subject to complex regulation, and this might contribute to its susceptibility to disruption. We found the *DTF6* allele in NGB5839 confers semi-dominant late flowering relative to the wild allele and increased *LF* expression, implying that it may represent a gain-of-function regulatory change.

The locus with the strongest effect after *DTF5a/HR* under SDs, *DTF1*, was also detected in an earlier study examining the F_2_ of this same cross (Weller *et al.*, 2012) and probably corresponds to the *E* locus originally distinguished by Murfet (1971). This is the only one of the classical pea loci not yet characterized at the molecular level. *DTF1* was in fact the strongest influence on flowering under LDs (PVE 19% for DTF, 31% for NFI) and its relative contribution was very similar in SDs (Table 1). Our results also capture the distinctive phenotype previously reported for the derived, domesticated early-flowering allele, in which early initiated flower buds abort at an early stage of growth, particularly under LD conditions (reflected in a greater PVE for NFI than DTF). Fine mapping in an advanced-generation segregating progeny located it very close to a previously unreported *FT* gene *FTa3* (Fig. 1).

The fourth locus *DTF3* had only a minor contribution to early flowering, but again under both photoperiod conditions (Table 1). Natural variation for flowering time has not previously been reported in this genomic region, but it has been implicated as a target of post-domestication selection (Siol *et al.*, 2017). An examination of candidates in this region showed that the QTL co-locates with *FTa1*/*GIGAS*, a gene which is known to to play a central role in promoting the transition to flowering in pea and Medicago (Hecht *et al.*, 2011; Laurie *et al.*, 2011).

The fifth locus *DTF5b* clearly represented the effects of Mendel’s *LE* gene which was segregating in the population (Fig. 1). This locus had a specific effect on flowering time, but only in LDs, and its lack of effect on flowering node indicates an effect on plant growth rate but not developmental timing. *LE* is well known as a gibberellin biosynthesis gene (Lester *et al.*, 1997), and previous characterizations of the *le-3* mutation in an isogenic comparison (Hecht *et al.*, 2007) and of a different mutant allele *le-1* (Murfet and Reid, 1987) reached similar conclusions about its effects on flowering.

It is curious that among the four loci with a developmental influence on flowering (i.e. excluding *DTF5b/LE*), three are likely to represent gain-of-function alleles of genes in the *FT/TFL1* family. The importance of these genes in crop phenological adaptation is now widely recognized (Eshed and Lippman, 2019; Gaudinier and Blackman, 2020), and several examples are known where apparent gain-of-function alleles at *FT* loci confer early flowering, and at least partially over-ride normal environmental constraints on the expression of the gene. For example, a single nucleotide polymorphism (SNP) and a small deletion in the promoter of the maize *FT* homologue *ZCN8* contribute to elevated expression and earlier flowering under LD conditions at high latitudes (Guo *et al.*, 2018). In wheat, a retroelement insertion in the promoter of the *FT* homologue *VRN-B3a* is associated with elevated expression and vernalization-independent early flowering (Yan *et al.*, 2006), whereas in barley, the same effect is associated with increased copy number of the *FT1* gene (Nitcher *et al.* 2013). Examples in legumes include the *DTF3a* locus in chickpea (Ortega *et al.*, 2019) and the *DTF6a* locus in lentil (Rajandran *et al.*, 2022), both of which map to *FTa1* orthologues and are associated with effects on their expression. In both species, dominant-early alleles appear to have had a key role in relaxing requirements for LDs and/or vernalization that have enabled spread to low-latitude regions in south Asia and Africa. In Medicago, induced transposon insertions within *FTa1* and in its 3ʹ-flanking sequence result in elevated *FTa1* expression and early flowering, and epigenetic repression at *FTa1* mediated by the polycomb gene *VRN2* is important to prevent its expression in the absence of vernalization (Jaudal *et al.*, 2013, 2016).

These examples suggest complex regulation around the *FTa1* gene that may include one or more repressive influences and both genetic and epigenetic effects. It is possible that this may not be unique to the *FTa1* gene, and in narrow-leafed lupin (*Lupinus angustifolius*) a similar de-repression of a paralogous gene *FTc1* is associated with promoter deletions and vernalization-independent early flowering (Nelson *et al.*, 2017). It is therefore plausible that similar mechanisms may operate in regulation of other genes in the wider pea *FT* family including *FTa3* and *LF*. Future exploration of sequence diversity, finer mapping, and more detailed analyses of transcription in the genomic regions around these candidate genes may help identify candidate causal polymorphisms and clarify relevant molecular evolution. It may also reveal distinct regulatory characteristics and/or conditional phenotypic expression that could help explain the basis for their selection. However, a comprehensive picture will await revised genome releases that feature more complete sequences around the genes and across the regions of interest.

Recent genomic analyses have clarified phylogenetic relationships within the genus *Pisum*, and strengthened the case for an independent domestication of the Ethiopian cultivated form *P. abyssinicum* (Kreplak *et al.*, 2019) from a distinct group of wild *P. s. elatius*. In keeping with their distribution at low latitudes, *P. abyssinicum* accessions are able to flower in short photoperiods (Weller *et al.*, 2012), and it will be of interest in future to determine the extent to which this adaptation might share a common genetic basis with that described here for *P. sativum*.

## Supplementary data

The following supplementary data are available at *JXB* online.

Table S1. Details of gene-based anchor markers used.

Table S2. Details of advanced-generation segregating populations.

Table S3. Details of primers used for gene expression.

Table S4: Summary of markers used for construction of high-density consensus map.

Table S5. Linkage map and associated sequence.

Table S6. Details of gene accessions and methods used for phylogenetic analysis

Fig. S1. Comparative analysis between the pea linkage map and *Pisum sativum* genome assembly.

Fig. S2. Comparative analysis between the pea linkage map and *Medicago truncatula*.

Fig. S3. Comparative analysis between the pea linkage map and *Cicer arietinum*.

Fig. S4. Comparative analysis between the pea linkage map and *Lens culinaris.*

Fig. S5. Comparative analysis between the pea linkage map and *Trifolium pratense*.

Fig. S6. LOD profiles for RN QTLs

Fig. S7. Flowering locus T phylogenic analysis in legumes.

erac132_suppl_Supplementary_Tables_S1-S2_S4-S5_Figures_S1-S7

erac132_suppl_Supplementary_Table_S3

erac132_suppl_Supplementary_Table_S6

## Data Availability

All data supporting the findings of this study are available within the paper and within its supplementary data published online.
