# Supplementary material for: The genetic architecture of flowering time changes in pea from wild to crop
Source: J Exp Bot. 2022 Apr 6;73(12):3978–90. doi: 10.1093/jxb/erac132 (PMC9238443; doi:10.1093/jxb/erac132)
Supplement: erac132_suppl_Supplementary_Tables_S1-S2_S4-S5_Figures_S1-S7 [file erac132_suppl_supplementary_tables_s1-s2_s4-s5_figures_s1-s7.pdf]

Supplemental Table 1: Details of gene-based anchor markers

		Primer sequence		Position	
**Ps**	**Gene**	**Forward**	**Reverse**	**Pea**	**Medicago**
**1**	***MTIC153***	TGCAACAAAAGAGGTATGAACTG	TGGGTCGGTGAATTTTCTGT		-
**1**	***BRC2***	AACCAGCTTAATTTTCTTCTTTG	CACTCAAAATTATGGAATTTCAGG	Psat1g061800	Medtr6g017055
**1**	***FTa3***	TTGTTCTTGGAGCTGTAATTGG	CCTCAAATTTGGGTTACTAGGG	Psat1g096760	Medtr6g033040
**1**	***NT6083***	CGTGTTTTTCTGAGTTGACTTCC	TGTATACAGGGCAAACTCCTTG	Psat1g096960	Medtr6g034195
**1**	***MLO1***	TGGCTCTTAGGCATGGATTT	TTGTGCATCATGTCCTGGAG	Psat1g099840	Medtr6g033330
**1**	***COP13***	ATAAAAGTTGATATGGGAGAAAGA	CCAATGCAGGCACTCATA	Psat1g084880	Medtr6g023350
**1**	***RNAhel***	GGGTTTGGTAGGTTTGGTAGAGG	GCATGTGCTATTTTTCTTCACTC	Psat4g086120**	Medtr6g056080
**1**	***CABB***	AGGATCTTCTTGCCTGATGG	CTTGCTTAGACCAAAAGGATCA	Psat1g111960	Medtr6g060175
**1**	***Ga20ox***	GACCAACTTTTTAAGAAAAGCA	TCTCCCATTTGAAAGAGCCTA	Psat1g113960	Medtr6g464620
**1**	***AGO1***	TTACTCCCATGTCATCCTTGG	CAAGCATTAAAGAACCAGCAAG	Psat1g122920	Medtr6g477980
**1**	***FULa***	AACCTAGTAGCTCTCACCGTAA	TTATATTATGGTGTTTGATTGATGA	Psat1g126560	Medtr2g461760
**1**	***APRL***	TGGGATGCTTCCTATTGGTT	TGAACATGGTCTGAAAATCTCAC	Psat1g130120*	Medtr6g029240
**1**	***BFT***	GGCCAATTTTGCTGATGACT	TTTGACCACACTTGGTTCAAC	Psat0s3447g0040	-
**3**	***FTa1***	GGACGTGAGCAAAACGACAT	TTGAGTAGTACCAGCACACACT	Psat3g090720	Medtr7g084970
**3**	***FTc***	CATTGGGATGTTAAAATGGTG	TGGGAAAGAGTTGCAAGATG	Psat3g091040	Medtr7g085040
**5**	***HR***	ACTAACACTTTATTGGCAAGTG	GCGGAAAGTATCGTCATTTTG	Psat5g037560	Medtr3g103970
**5**	***LE***	TGTCGTGCAATATGATGAAACC	CGGCCCATTTGATATCTTCC	Psat5g299720	Medtr2g102570
**6**	***A***	-	-	Psat6g060480	Medtr1g072320
**6**	***LF***	GGTCCCTCTTTACCCTGGTATT	TGATCTGCAGGAAAACAATAAA	Psat6g013960	Medtr1g060190
**6**	***TMP***	CACCCACAAATCCCTCTTCC	AACAGCCCATGATTTAGCGG	Psat6g017040	Medtr1g017450
**6**	***ERMP***	CATCAGATTGGGTGTCGTCC	AGACCACCACCTGAGTATTCC	Psat6g025120	Medtr1g013660
**6**	***CYTB7***	TCAAGGAGGCTCTGAATCGG	CCTGAATGGTGTTTGCATTGC	Psat6g030080	Medtr1g011880
**6**	***JMJ***	CTAGAGTGAAGTGTAATTGTAAG	TGCCAGAATAAGGAAAATGGAG	Psat6g052840	Medtr1g078070
**7**	***DUF***	GTGGCAAGCTCATCCAAAAT	CTACCGCCCAAGATTGTTTC	Psat7g132880	Medtr4g083440

* Nearest gene
** Uncertain mapping in genome

Supplemental Table 2: Summary of markers used for construction of high density consensus map

Table showing number of markers used in the construction of the skeleton map before and after marker exclusion.

	LGI	LGII	LGIII	LGIV	LGV	LGVI	LGVII	Total
**Markers in skeleton map**	109	139	159	124	150	108	118	905
**Markers added**	416	508	700	578	408	512	593	3692
**Combined Markers**	525	647	857	702	558	620	711	4599
**Size (cM)**	182.31	249.86	279.49	217.34	288.53	182.70	216.73	1616.96
**Density (marker/cM)**	2.88	2.59	3.07	3.94	2.43	3.39	3.28	3.08 (average)
**Number of gaps (> 10cM)**	0	0	0	0	0	0	0	0
**Segregation distortion % (P = <10)**	0	11.51	15.92	12.1	0	6.31	44.07	12.84 (average)

Supplemental Table 4: Details of advanced generation segregating populations

A. Advanced generation segregating populations used for fine mapping

**Advanced pop. for**	**Generation**	**DTF1**	**DTF3**	**DTF5a**	**DTF5b**	**DTF6**	**n**
DTF1	F5	H	W	W	D	D	32
DTF3	F4	W	H	W	W	W	45
DTF6	F4	D	W	W	H	H	85

W: homozygous wild allele
D: homozygous domesticated allele
H: heterozygous

B. Advanced generation segregating populations used for expression analysis

**Advanced pop. for**	**Generation**	**DTF1**	**DTF3**	**DTF5a**	**DTF5b**	**DTF6**
DTF1	F6	D	W	W	D	D
		W	W	W	D	D
DTF3	F5	W	W	W	W	W
		W	D	W	W	W
DTF6	F5	D	W	W	W	W
		D	W	W	W	D

W: homozygous wild allele
D: homozygous domesticated allele
H: heterozygous

Supplemental Table 5: Details of primers used for qRT-PCR

Gene	Primer 1	Primer 2	Temp (ºC)
ACT	GTGTCTGGATTGGAGGATCAATC	GGCCACGCTCATCATATTCA	59
LF (TFL1c)	CAGACATTCCAGGGACAACAG	AAATAAGCAGCAGCAACAGGG	60
FTa1	GCCCAAGCAACCCTACTTTT	CCATCCTGGAGCGTAAACCC	60
FTc	GATATTCCAGCCACAACAAGC	TTATGACGCCACTCTGGAGCAA	62
FTa3	GGATCCAGATGCACCTAGCC	CGATGAATCCCCATCAACG	60

Supplemental Figure 1: Comparison of pea linkage map and *Pisum sativum* genome assembly.

Syntenic regions highlighted in colour

Linkage map	*P. sativum* genome assembly (v1a) Ps2	Ps6	Ps5	Ps4	Ps3	Ps1	Ps7	Total	Mapped	Unmapped	Syntenic %)
LGI	471	3	0	3	3	8	7	524	495	29	95
LGII	0	571	6	6	4	7	4	638	598	40	93
LGIII	3	7	761	8	5	10	8	854	802	52	93
LGIV	5	0	5	604	3	4	9	701	630	71	94
LGV	1	4	6	5	474	10	3	537	503	34	92
LGVI	1	5	9	11	0	530	4	606	560	46	93
LGVII	3	4	12	10	6	14	656	709	705	4	92
Total	484	594	799	647	495	583	691	4569	4293	276	-
Syntenic (%)	97	96	95	93	96	91	95	-	-	-	93

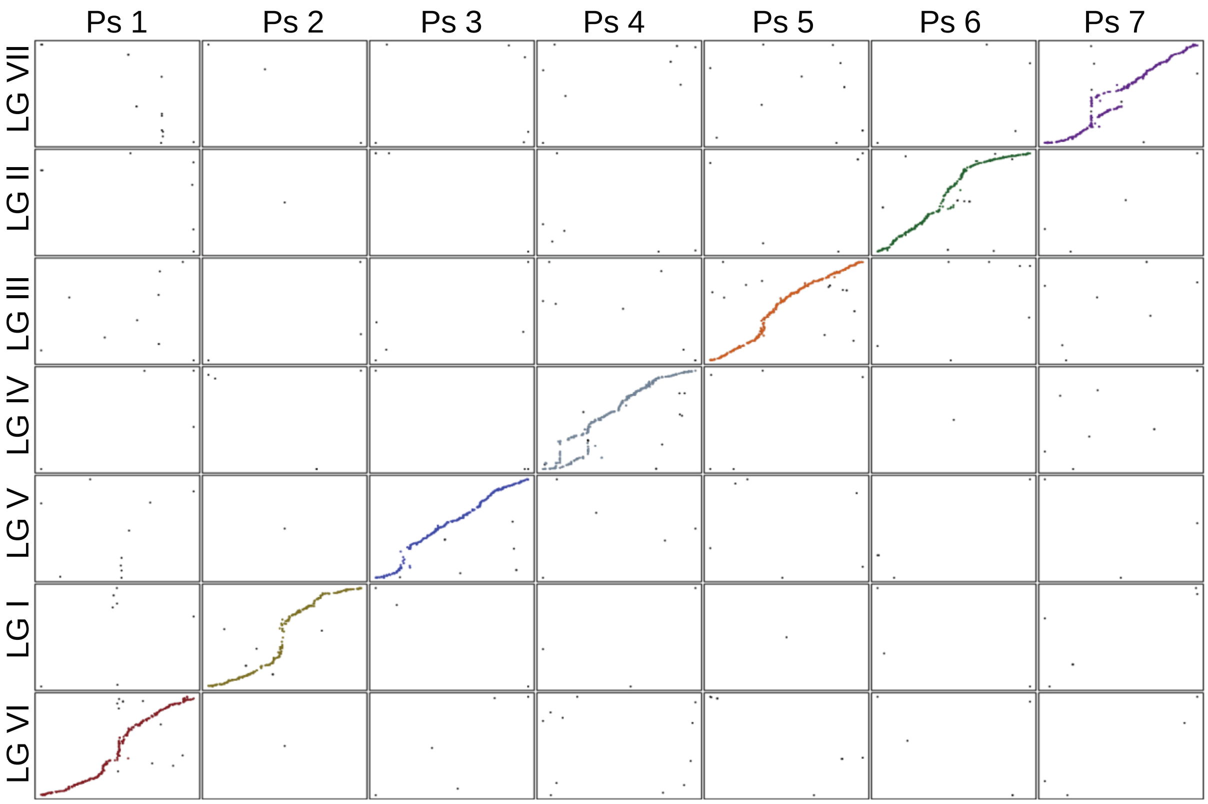

Supplemental Figure 2: Comparison of pea linkage map and *Medicago truncatula* genome.

Syntenic regions highlighted in colour

		M. truncatula genome assembly (v4.0)											
		Mt1	Mt2	Mt3	Mt4	Mt5	Mt6	Mt7	Mt8	Total	Mapped	Unmapped	Syntenic (%)
Linkage map	LGI	31	24	23	27	276	13	21	22	524	437	87	60
	LGII	364	22	27	32	31	16	29	16	638	537	101	62
	LGIII	49	127	382	44	23	28	30	32	854	715	139	60
	LGIV	41	39	30	213	42	21	25	167	701	578	123	59
	LGV	22	17	31	32	16	18	290	24	537	450	87	60
	LGVI	28	202	37	46	21	103	38	24	606	499	107	46
	LGVII	37	31	22	284	35	12	31	145	709	597	112	62
	Total	572	462	552	678	444	211	464	430	4569	3813	756	-
	Syntenic (%)	58	57	60	63	63	28	58	62	-	-	-	58

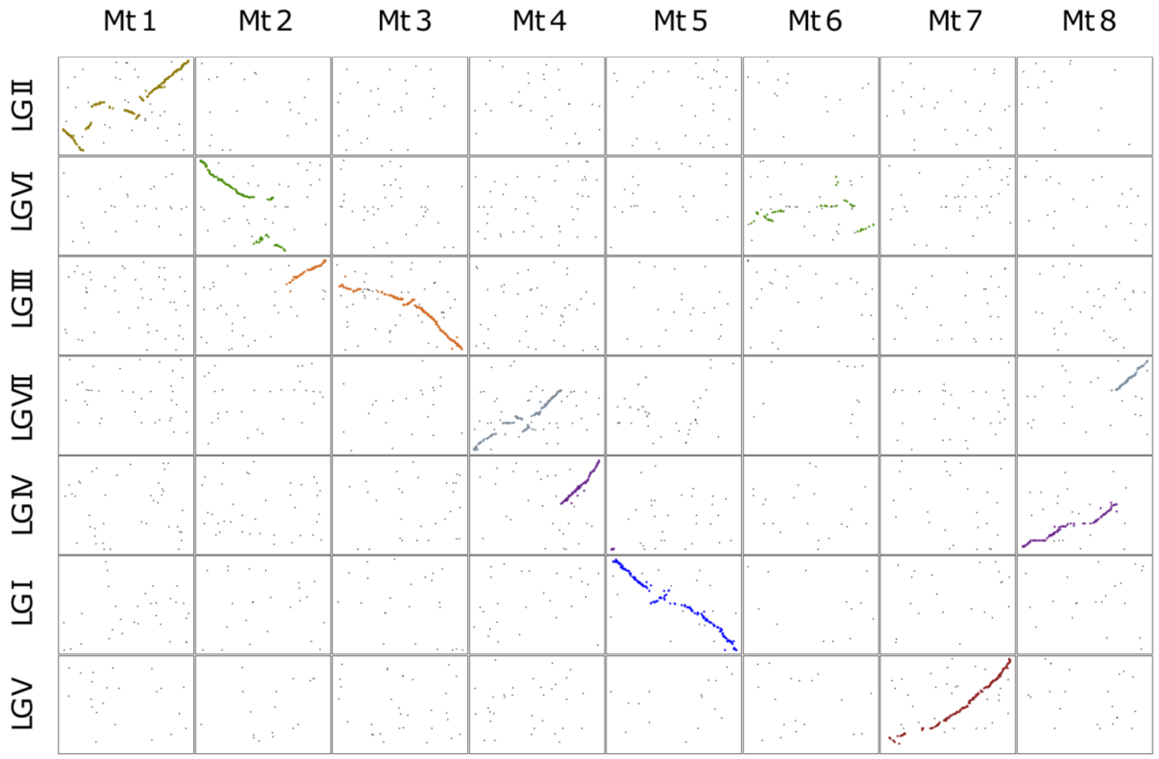

Supplemental Figure 3: Comparison of pea linkage map and *Cicer arietinum* genome.

Syntenic regions highlighted in colour

		C. arietinum genome assembly (v2.0)											
		**Ca1**	**Ca2**	**Ca3**	**Ca4**	**Ca5**	**Ca6**	**Ca7**	**Ca8**	**Total**	**Mapped**	**Unmapped**	**Syntenic (%)**
**Linkage map**	**LGI**	36	145	21	31	36	34	38	107	524	448	76	50
	**LGII**	28	27	34	348	26	45	29	16	638	553	85	57
	**LGIII**	116	32	27	61	343	77	46	20	854	722	132	55
	**LGIV**	47	28	33	44	42	38	328	33	701	593	108	51
	**LGV**	25	30	265	33	33	38	27	14	537	465	72	54
	**LGVI**	196	57	52	30	39	55	48	40	606	517	89	34
	**LGVII**	37	35	26	45	45	367	33	17	709	605	104	55
	**Total**	485	354	458	592	564	654	549	247	4569	3903	666	-
	**Syntenic (%)**	47	46	51	56	52	53	53	43	-	-		51

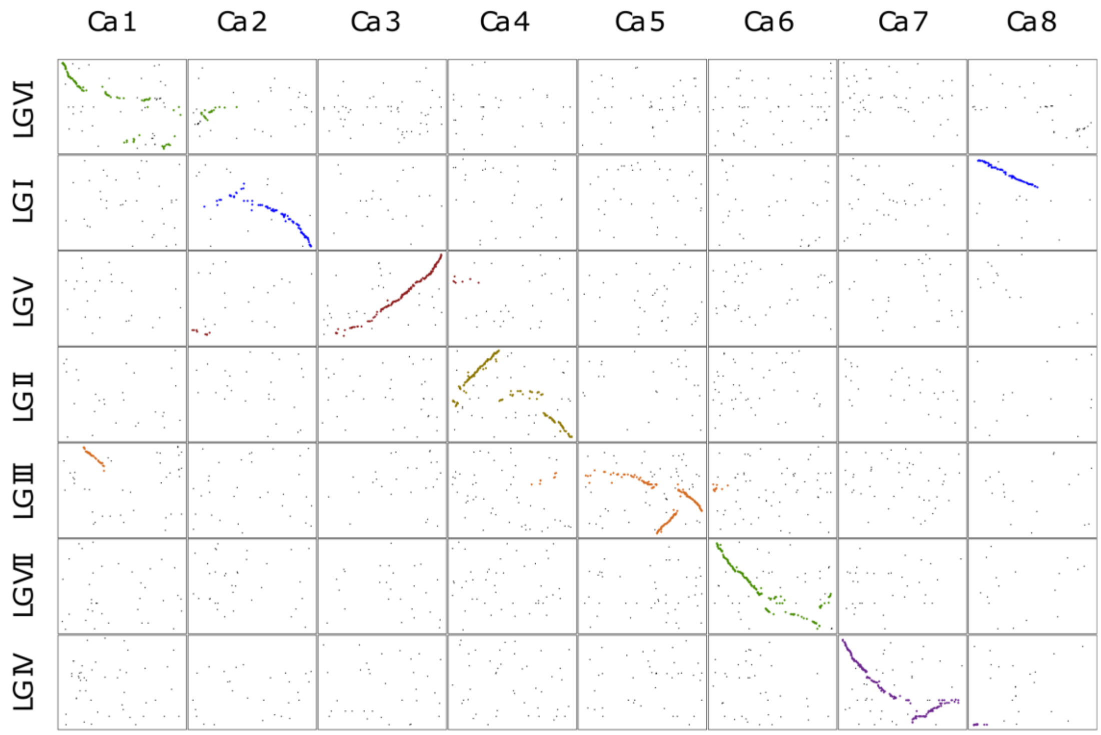

Supplemental Figure 4: Comparison of pea linkage map and *Lens culinaris* genome.

Syntenic regions highlighted in colour

		***L. culinaris* genome assembly (v1.0)**										
		**Lc1**	**Lc2**	**Lc3**	**Lc4**	**Lc5**	**Lc6**	**Lc7**	**Total**	**Mapped**	**Unmapped**	**Syntenic (%)**
**Linkage map**	**LGI**	28	2	7	3	189	1	2	524	232	292	74
	**LGII**	243	0	6	0	47	1	2	638	297	341	83
	**LGIII**	2	63	258	2	11	8	2	854	346	508	72
	**LGIV**	1	2	3	2	17	3	233	701	261	440	64
	**LGV**	5	1	5	5	2	194	1	537	213	324	60
	**LGVI**	2	212	0	2	1	1	2	606	220	386	75
	**LGVII**	3	3	7	258	3	2	2	709	278	431	74
	**Total**	284	283	284	272	270	210	244	4569	1847	2722	-
	**Syntenic (%)**	78	72	70	73	71	59	67	-	-	-	72

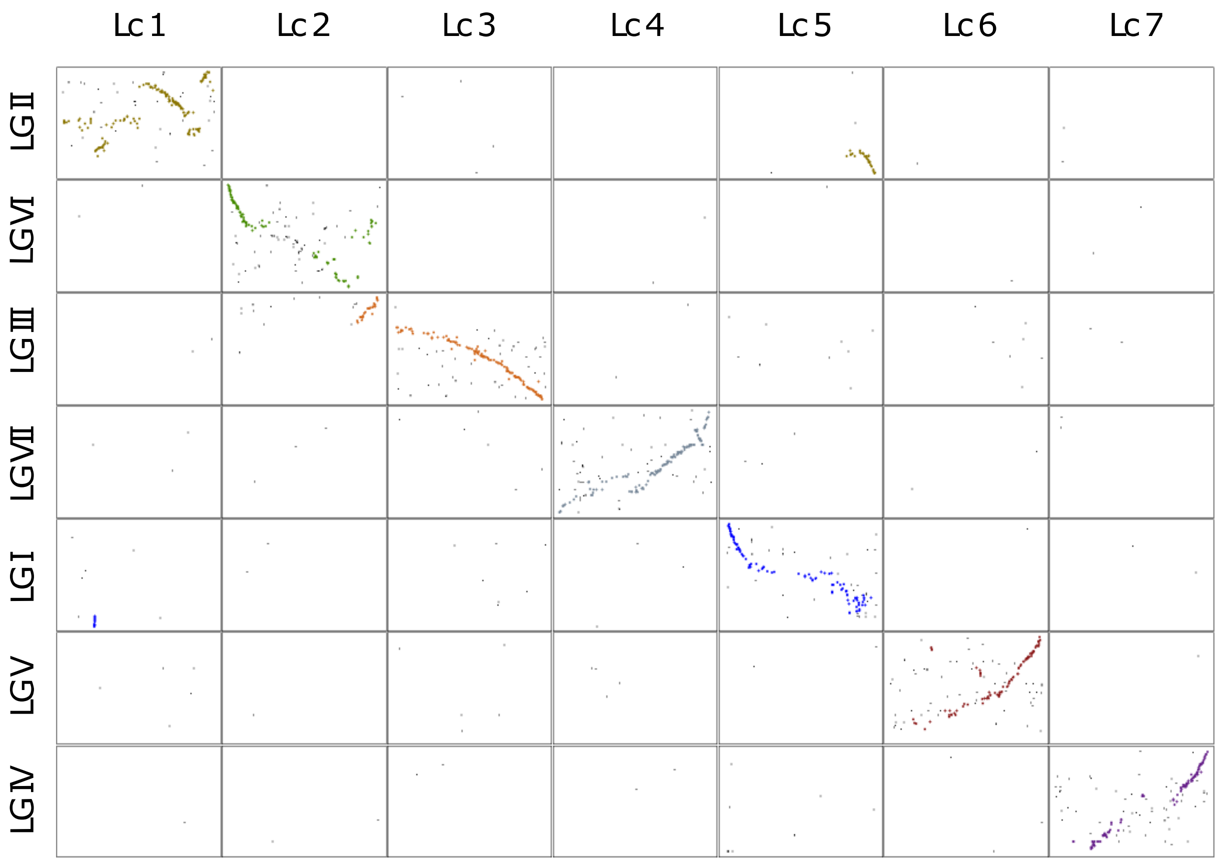

Supplemental Figure 5: Comparison of pea linkage map and *Trifolium pratense* genome.

Syntenic regions highlighted in colour

		T. pratense genome assembly (v2.0)										
		Tp1	Tp2	Tp3	Tp4	Tp5	Tp6	Tp7	Total	Mapped	Unmapped	Syntenic (%)
Linkage map	LGI	50	137	69	82	31	46	45	524	460	64	23
	LGII	251	41	56	49	24	57	35	638	513	125	31
	LGIII	53	90	159	76	33	52	250	854	713	141	36
	LGIV	75	130	62	97	100	56	59	701	579	122	35
	LGV	53	48	58	33	23	186	48	537	449	88	31
	LGVI	62	135	59	58	38	40	92	606	484	122	10
	LGVII	54	79	230	128	19	45	61	709	616	93	43
	Total	165	89	128	216	114	183	248	4569	3814	755	-
	Syntenic (%)	28	32	37	30	24	31	28	-	-	-	31

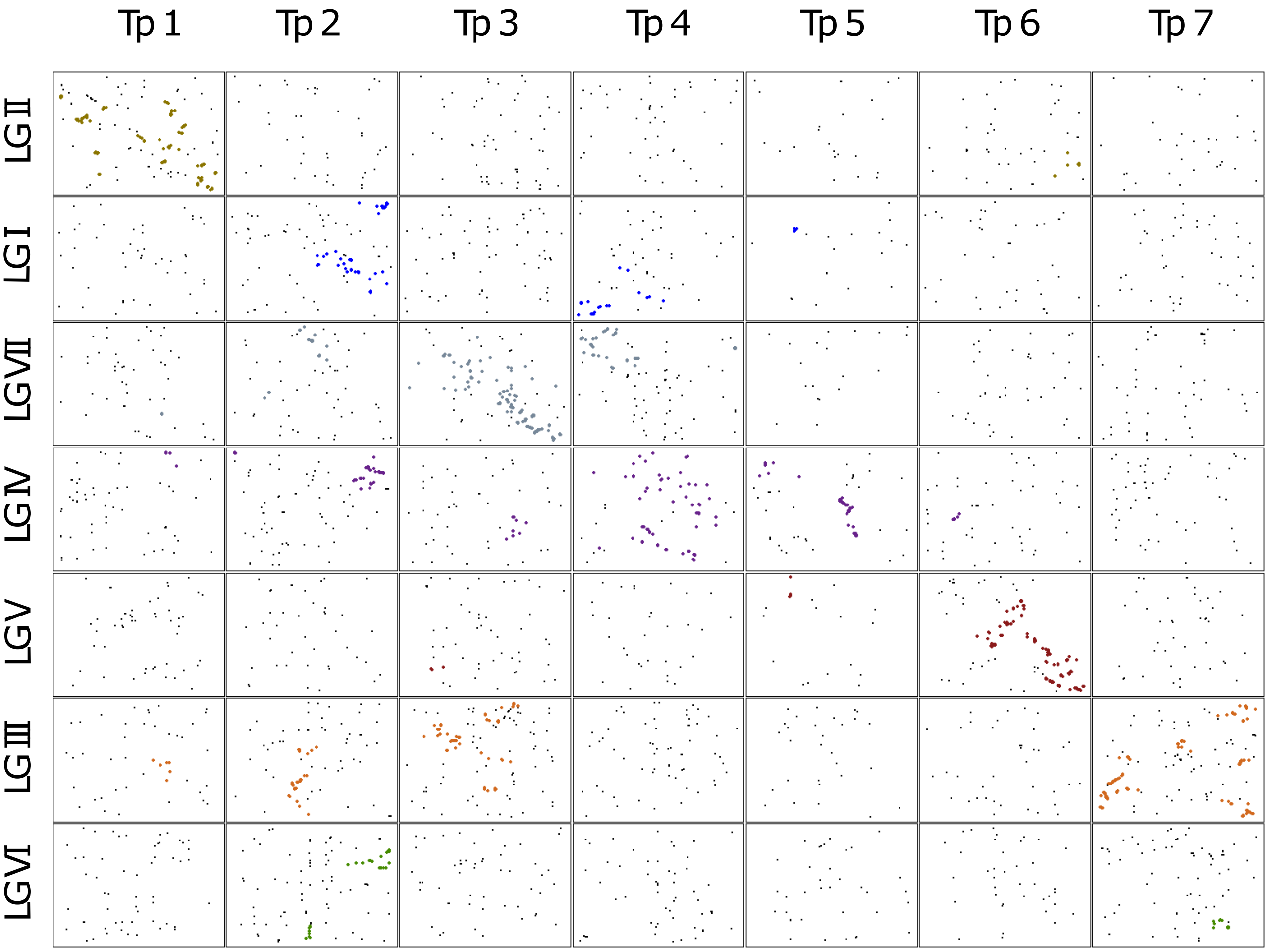

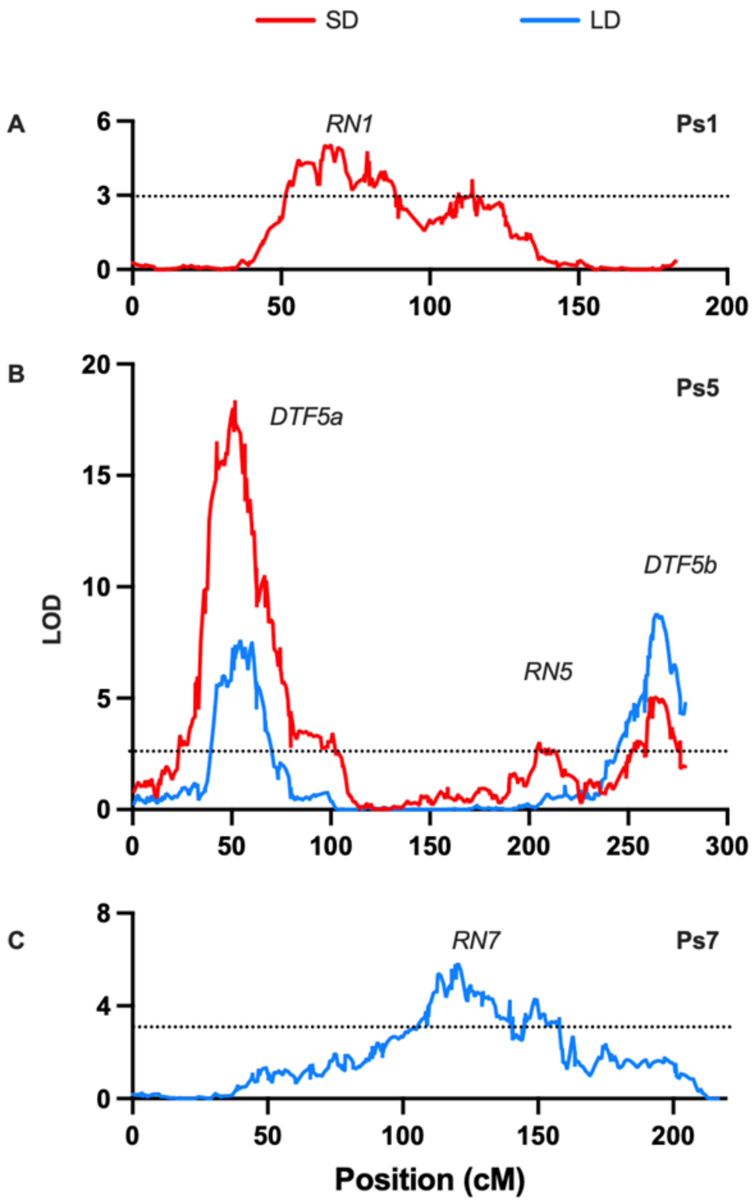

Supplemental Figure 6: LOD profiles for RN QTL

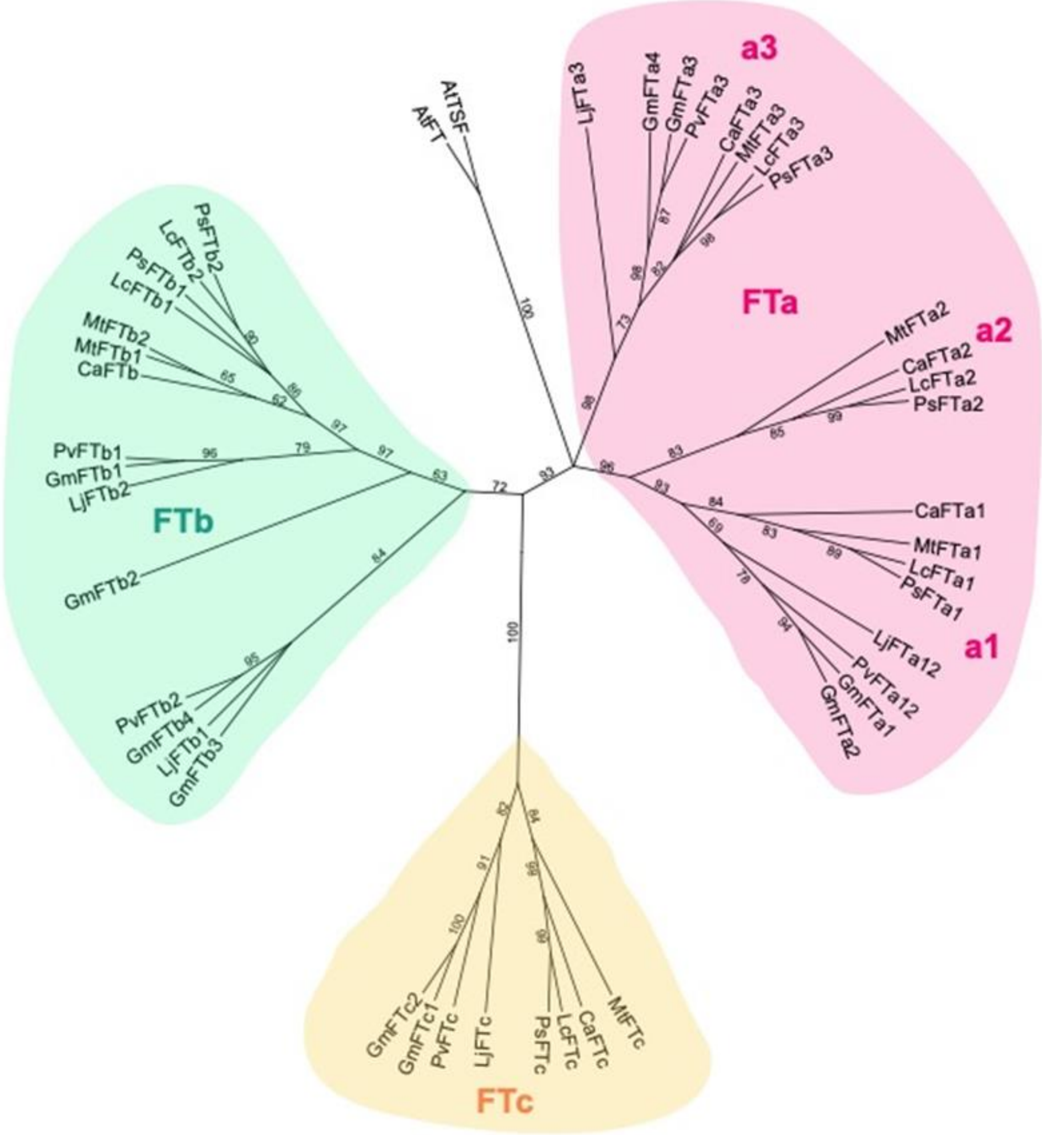

Supplemental Figure 7: Flowering locus T phylogenic analysis in legumes
